# *PTCH2* is not a strong candidate gene for gorlin syndrome predisposition

**DOI:** 10.1007/s10689-021-00269-7

**Published:** 2021-06-25

**Authors:** Miriam J. Smith, D. Gareth Evans

**Affiliations:** Manchester Centre for Genomic Medicine, St Mary’s Hospital, Manchester Academic Health Sciences Centre, Division of Evolution and Genomic Science, Faculty of Biology, Medicine and Health, The University of Manchester, Manchester, M13 9WL UK

**Keywords:** Gorlin syndrome, Basal cell carcinoma, BCNS, NBCCS, *PTCH2*

## Abstract

A number of case/family reports have proposed *PTCH2* as a putative Gorlin Syndrome (GS) gene, but evidence to support this is lacking. We assessed our cohort of 21 *PTCH1/SUFU* negative GS families for *PTCH2* variants and assessed current evidence from reported cases/families and population data. In our *PTCH1/SUFU* variant negative GS cohort (25% of total), no pathogenic or likely pathogenic *PTCH2* variants were identified. In addition, none of the previously published *PTCH2* variants in GS families/cases could be considered pathogenic or likely pathogenic using current guidelines. The absence of clear pathogenic variants in GS families and the high frequency of Loss-of-function (LoF) variants in the general population, including the presence of homozygous LoF variants without a clinical phenotype, mean that it is untenable that *PTCH2* is a GS gene. *PTCH2* should not be included in panels for genetic diagnosis of GS.

## Introduction

Gorlin syndrome (GS), also known as basal cell nevus syndrome (BCNS) and nevoid basal cell carcinoma syndrome (NBCCS) is an autosomal dominant tumour predisposition syndrome which predisposes affected individuals to the development of odontogenic jaw keratocysts and multiple basal cell carcinomas as well as a number of dysmorphic features and congenital abnormalities. Pathogenic variants in the *PTCH1* gene are the most common genetic abnormalities associated with GS [1]. Occasionally, variants in *SUFU* have been identified as the cause of classic GS with a much higher risk of infantile medulloblastoma (20–30% risk) than *PTCH1* related GS (1–2% risk) and there is no real evidence for keratocysts in *SUFU-*associated GS [2, 3]. The identification of *PTCH2* in 1999 [4] led to speculation that this may also be a cause of GS. Although no germline pathogenic variants were found in the initial report, somatic mutations in a medulloblastoma and a basal cell carcinoma were found [4]. It was not until nine years later that two reports from China suggested that missense variants in *PTCH2* could be linked to GS [5, 6]. Xu and Li reported the variants c.323 T > C and c.1319C > T [6]. On recent review of the nomenclature, we noted that the variants are now annotated as c.311 T > C, p.Leu104Pro and c.1307C > T, p.Ala436Val. The first variant, p.Leu104Pro, is now known to occur in 73/282696 alleles in gnomAD data (frequency = 0.000258) (including 1 homozygote) (https://gnomad.broadinstitute.org/transcript/ENST00000372192?dataset=gnomad_r2_1) accessed December 21st 2020. The second variant, c.1307C > T, p.Ala436Val, is found in 18/280892 alleles on gnomAD (frequency = 0.000064). Another variant, reported by Fan et al. in one large family, is p.Arg719Gln [5]. This variant is found in 8/281616 alleles on gnomAD (frequency = 0.000028). Thus, these three variants, summarised in Table 1, have all been found multiple times in the gnomAD cohort and are too common to be a relatively minor genetic contributor to GS.

**Table 1:** Previous reports of germline *PTCH2* variants associated with GS phenotype

	Family or single case	Meets criteria	Variant	ACMG^a^ classification	gnomAD frequency
Casano et al. [7]	Family	No	c.3347C > T; p.(Pro1116Leu)	3 (Uncertain significance)	2/247660 (8.08e−6)
Fujii et al. [8]	Case	No	c.1172_1173delCT; p.Ser391^a^	2/3 (Likely benign/uncertain significance)	64/282846 (2.26e−4) (includes 1 homozygote)
Fan et al. [5]	Family	Yes	c.2157G > A; p.Arg719Gln	3 (Uncertain significance)	8/281616 (2.84e−5)
Xu et al. [6]	Unclear	Unclear	c.311 T > C, p.Leu104Pro	2 (Likely benign)	73/282696 (2.58e−4) (includes 1 homozygote)
Xu et al. [6]	Unclear	Unclear	c.1307C > T, p.Ala436Val	3 (Uncertain significance)	18/280892 (6.41e−5)

^a^American College of Medical Genetics guidelines for variant classification[11]

Since 2008, there have been two further reports of a family [7] and a single case [8] of *PTCH2*-associated GS. However, while GS features are described, these individuals did not meet clinical criteria.

Despite a report of a healthy woman in her late 30 s with a homozygous truncating variant, but no GS features, and with a daughter who also has no GS features [9], review articles still cite *PTCH2* as a cause of GS [10]. We have therefore assessed the potential contribution of *PTCH2* to GS in our own data, by assessing the gene in the germline of GS families who have previously been found negative for pathogenic *PTCH1* and *SUFU* variants. We have also assessed the frequency of loss-of-function variants in gnomAD data.

## Materials and methods

### Patient material

A total of 86 unrelated individuals were identified as meeting clinical diagnostic criteria for Gorlin syndrome and as having undergone previous clinical genetic screening for pathogenic *PTCH1* variants using a combination of Sanger sequencing and multiplex ligation-dependent probe amplification (MLPA). For eight individuals with RNA samples available RNA analysis had also been undertaken. Lymphocyte DNA was available for further genetic analysis on all 27 people who tested negative for *PTCH1* variants. Routine screening was carried out through the diagnostic service at the West Midlands Genetics Laboratory, UK. Research analysis was carried out with ethical approval by the National Research Ethics Service Committee North West 7 (10/H1008/74).

### Mutational analysis

DNA was purified using Zymo Genomic DNA Clean and Concentrator columns (Zymo Research Irvine, CA, USA) and exome sequencing analysis was carried out on lymphocyte DNA in-house using an Illumina HiSeq, or by BGI-Tech solutions (Hong Kong) Co Ltd, using paired-end 100 bp and sequenced to 50x. *PTCH1*, *SUFU* and *PTCH2* variants were annotated using VarSeq software (Golden Helix Inc, MT, USA) and variant pathogenicity was assessed using American College of Medical Genetics (ACMG) guidelines [11].

All variants detected by exome sequencing were subsequently validated by Sanger sequencing. Selected regions were amplified by PCR using GoTaq G2 PCR mastermix and products were purified using AxyprepMag PCR cleanup beads (Axygen Biosciences, CA, USA). DNA sequencing was performed using BigDye® Terminator v3.1 Cycle Sequencing Kit (ABI, Life Technologies, CA, USA). Sequencing PCR products were purified using Axyprep Mag DyeClean beads (Axygen Biosciences, CA, USA) and sequence analysis was performed using an ABI 3730xl DNA Analyzer (ABI, Life Technologies, USA).

### Copy number analysis

Copy number analysis for detection of large deletions in *PTCH1* used the PTCH1 SALSA MLPA probemix P067-B1-0512 PTCH1v13 (MRC-Holland, The Netherlands). The large *SUFU* deletion was identified using an in-house assay designed in the laboratory of Dr Christian Beetz, and carried out as previously described [2].

## Results

Lymphocyte DNA from a total of 86 unrelated individuals meeting clinical criteria for GS was screened for pathogenic *PTCH1* variants using Sanger sequencing and, where material was available, RNA analysis [2]. This identified pathogenic *PTCH1* variants in 59 people (68.6%). Exome sequencing was carried out on the remaining 27 *PTCH1* negative individuals. This identified 2 more *PTCH1* variants that had been missed on historical clinical Sanger sequencing, perhaps due to the use of legacy transcript annotation. In addition, we have previously reported three families from this cohort with pathogenic *SUFU* variants, found using a combination of sequencing and copy number analysis [2]. A fourth family has subsequently been identified with a pathogenic *SUFU* variant. Thus 65/86 families (75.6%) with GS have an identifiable *PTCH1* or *SUFU* causative variant. No pathogenic (class 5) or likely pathogenic (class 4) *PTCH2* variants were identified in anyone from this cohort, according to the ACMG guidelines [11].

Assessment of *PTCH2* loss-of-function variants identified in the gnomAD cohort, found 355 loss-of-function (frameshift, nonsense and canonical splice-site) variants in the canonical *PTCH2* isoform (ENST00000372192.3), equivalent to 1 in 324 individuals, including a single case of a female who was homozygous for p.Ser391*, the same variant identified in the case reported by Fujii et al. [8] and more recently in a report of a Korean patient who also did not fulfil clinical diagnostic criteria for Gorlin syndrome [12].

In addition, 1 in 44 individuals on gnomAD were found to carry a rare missense (< 0.0002 population allele frequency) and 1 in 17 carried a missense with a population allele frequency of 0.0002–0.01. Therefore, there is a 2–6% chance of finding at least a class 3 variant of uncertain significance (VUS) when screening *PTCH2*.

## Discussion

Given that the estimated birth incidence of GS is around 1 in 15,000 and that over 75% of these are accounted for by *PTCH1* and *SUFU*, only the equivalent of around 1 in 60,000 can be due to other genes. Despite a rate of loss-of-function variants of 1 in 324 in the general population, *PTCH2* did not account for any of our 21 clinically affected unfound families. In addition to our 21 families and the 22 families from the original *PTCH2* gene discovery report [4], it is likely that many more GS families have been screened for *PTCH2* variants, yet only one truncating variant has been reported and this is present at a high frequency in gnomAD and has also been seen in homozygous form. It is possible, even likely, that over 300 people have been screened and meaning that finding one variant is consistent with chance. None of the reported missense variants that are also frequent in gnomAD would be classified as pathogenic or likely pathogenic, according to ACMG guidelines. In particular, the c.311 T > C, p.Leu104Pro is classified as class 2 or lower due to frequency and homozygosity. It is therefore simply untenable that *PTCH2* is a *bone fide* GS predisposition gene and at most may act as a modifier of the phenotype. *PTCH2* should therefore not be included in panels to identify GS causing variants and individuals identified with a *PTCH2* variant incidentally should be reassured that it is likely to be of no particular consequence and is certainly not a risk for GS.

## Data Availability

Anonymised data are available from the authors on request.
